# A fungal-derived benzothiazole derivative overcomes osimertinib resistance in NSCLC by targeting SCD1

**DOI:** 10.1007/s13659-026-00645-9

**Published:** 2026-09-11

**Authors:** Boyin Li, Yuanyu Ke, Jinhua Zhu, Weisong Zhang, Mingjin Yang, Zhan Li, Mu Chen, Zhiling Liu, Jieyao Liu, Qiudi Deng, Peng Zhang, Xueping Lei

**Affiliations:** 1 Guangzhou Municipal and Guangdong Provincial Key Laboratory of Molecular Target & Clinical Pharmacology, The NMPA and State Key Laboratory of Respiratory Disease, College of Pharmacy & The Fifth Affiliated Hospital, Guangzhou Medical University, Guangzhou, 511436 People’s Republic of China; 2 The Second School of Clinical Medicine, Guangzhou Medical University, Guangzhou, 510260 People’s Republic of China; 3 GMU-GIBH Joint School of Life Sciences, The Guangdong-Hong Kong-Macao Joint Laboratory for Cell Fate Regulation and Diseases, Guangzhou Medical University, Guangzhou, 511436 People’s Republic of China; 4 Tobacco Research Institute of Chinese Academy of Agricultural Sciences, Qingdao, 266101 People’s Republic of China; 5 Anti-Drug Technology Center of Guangdong Province (National Anti-Drug Laboratory Guangdong Regional Center), Guangzhou, 510230 P.R. China

**Keywords:** 6-(2-hydroxyethyl) benzo[d]thiazol-4-ol (HBT), Osimertinib resistance, Non-small lung cancer, SCD1, Ferroptosis

## Abstract

Osimertinib resistance remains a critical factor for treatment failure in non-small cell lung cancer (NSCLC), making it of great clinical significance to develop novel agents that can overcome this resistance. Natural products are invaluable sources for anti-drug discovery. In this study, we first report that 6-(2-hydroxyethyl) benzo[d]thiazol-4-ol (HBT), a benzothiazole derivative isolated from the endophytic fungus *Aspergillus* sp. 1022LEF, effectively reverses osimertinib resistance both in vitro and in vivo. Our results demonstrate that HBT effectively inhibits the proliferation of osimertinib-resistant cells by inducing ferroptosis, a novel form of iron-dependent cell death driven by lipid peroxidation. This effect is partially dependent on stearoyl-CoA desaturase 1 (SCD1). Further mechanistic studies revealed that HBT acts as a novel SCD1 inhibitor by directly binding to SCD1 and promoting its degradation, thereby leading to lipid peroxidation and ferroptosis. In vivo experiments showed that HBT treatment significantly suppressed tumor growth in PC9-OR xenograft models, as evidenced by reduced tumor volume and weight. In summary, our study not only identifies HBT as a promising therapeutic candidate for overcoming osimertinib resistance in NSCLC but also suggest that SCD1 is a potential target for combating such resistance.

**Graphical Abstract:**

**Figure:**
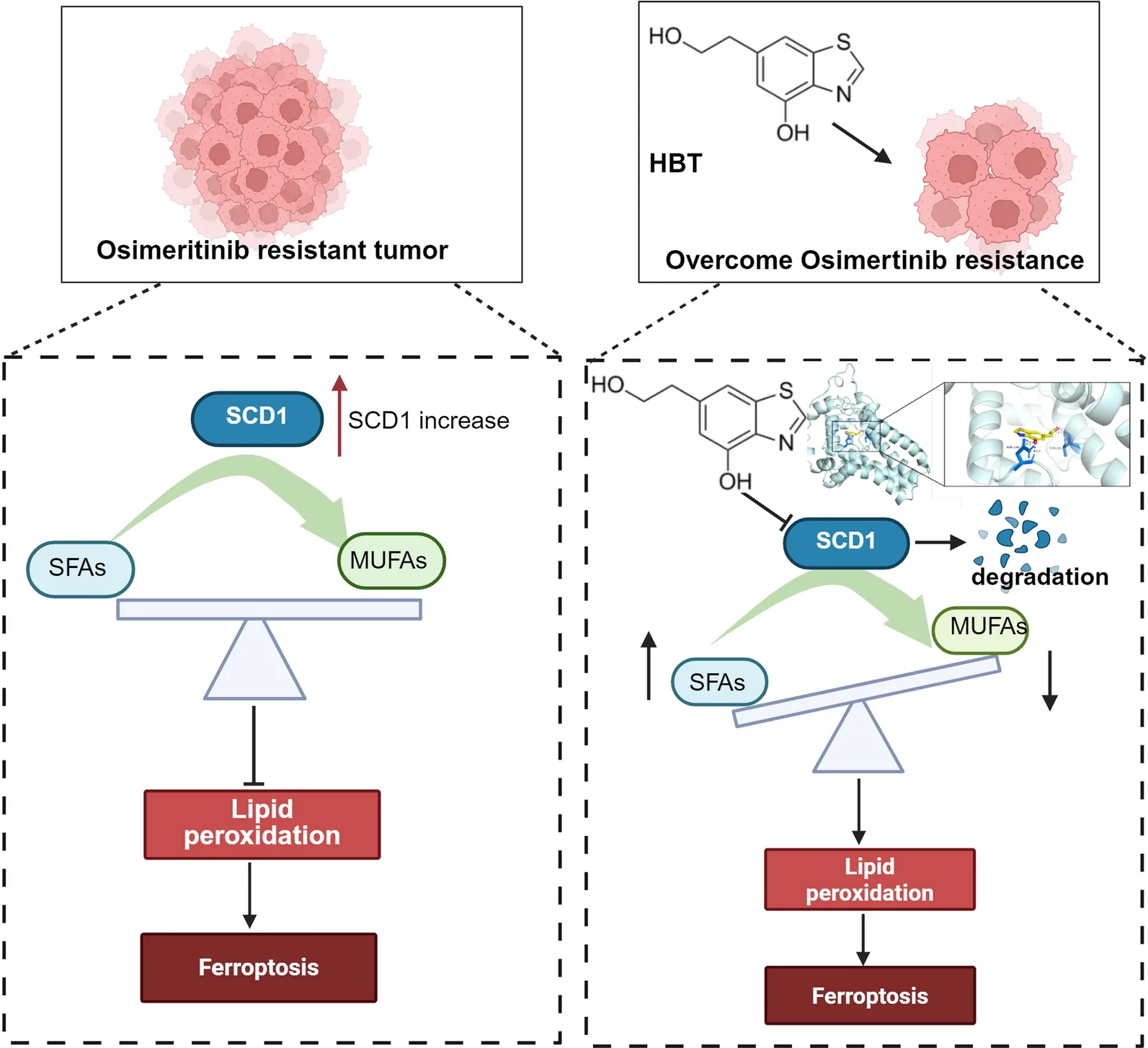

**Supplementary Information:**

The online version contains supplementary material available at https://doi.org/10.1007/s13659-026-00645-9.

## Introduction

Non-small cell lung cancer (NSCLC), account for approximately 85% of lung cancer, is one of most common cancers that characterized with high morbidity and mortality [1]. Since the introduction of the first epidermal growth factor receptor (EGFR) inhibitor in 1976, a series of these agents, from first- to third-generation, have been developed and applied in NSCLC therapy [2]. Osimertinib, an oral third-generation EGFR inhibitor, was specifically designed to target both EGFR-activating mutations and the T790M resistance mutation. While NSCLC patients treated with osimertinib show significant improvements in response rates and median progression-free survival, the majority patients inevitably develop acquired resistance, which limits the long‑term efficacy. And still now, there is not effective agent that can overcome osimertinib resistance. Therefore, it is essential to identify novel agents capable of overcoming osimertinib resistance [3, 4].

Numerous mechanisms contribute to osimertinib resistance and they can be classified into EGFR-dependent and EGFR-independent mechanisms [5]. EGFR-dependent mechanism primarily involves mutations such as C797S, L718Q, G719C, L792F/H/Y, G796S/R/D, and G724S. EGFR-independent mechanisms include oncogenic fusions, activation of bypass pathways (e.g., MET and HER2 amplification, TP53 mutation), and phenotypic transformations such as epithelial-mesenchymal transition (EMT). Additionally, activation of the PI3K/Akt and RAS-MAPK pathways, as well as AXL overexpression, have been implicated in resistance [6, 7]. Still now, there are approximately 40% underlying mechanism of osimertinib resistance is unclear. Although numerous therapeutic strategies targeting these mechanisms have been explored, the survival outcomes for patients with osimertinib-resistant NSCLC remain poor [8–10]. Thus, it is urgent and clinically desirable to develop potential therapeutic strategy that can overcome osimertinib resistance.

Natural products have long served as an invaluable reservoir for anticancer drug discovery, forming the basis of numerous clinically approved chemotherapeutic agents [11]. Prominent examples include plant-derived compounds such as vinca alkaloids, taxanes, and topoisomerase inhibitors, all of which remain cornerstones of modern cancer therapy. The structural diversity and favorable pharmacological properties of natural products continue to offer unique opportunities for the development of novel anticancer agents [12, 13]. Inspired by natural product-based drug discovery, our group has focused on identifying novel bioactive compounds from endophytic microorganisms. This effort led to the recent isolation of 6-(2-hydroxyethyl)benzo[d]thiazol-4-ol (HBT), a benzothiazole derivative from the fungus *Aspergillus* sp. 1022LEF, which demonstrated pronounced inhibitory effects against *S. frugiperda* [14]. However, whether HBT can overcome osimertinib resistance remains to be explored.

In this study, we identified HBT is a promising candidate for overcoming osimertinib resistance. HBT effectively reversed osimertinib resistance both in vitro and in vivo, with a favorable safety profile. Mechanistic investigations revealed that HBT acts as a novel stearoyl-CoA desaturase 1 (SCD1) inhibitor that induced ferroptosis in osimertinib-resistant PC9 (PC9-OR) cells. The pro-ferroptotic effect of HBT was antagonized by SCD1 overexpression and enhanced by SCD1 knockout. Furthermore, we demonstrated that HBT directly binds to SCD1 and promotes its protein degradation, thereby triggering ferroptosis and overcoming osimertinib resistance. Taken together, our study firstly identified HBT as an SCD1 inhibitor and implied that HBT might be developed into a potential agent for overcoming osimertinib resistance.

## Materials and methods

### Materials/Reagent preparation

6-(2-hydroxyethyl) benzo[d]thiazol-4-ol (HBT) was isolated from endophytic *fungus Aspergillus* sp.* 1022LEF* and its chemical structure was identified using LC/MS in our previous study [14]. osimertinib, Z-VAD-FMK, ferrostatin-1 (Fer-1), chloroquine (CQ) and necrostatin-1 (Nec-1) were products of TargetMol (Shanghai, China). Antibodies against SCD1(#2794), NCOA4 (#66849), SLC7A11 (#98051), GPX4 (#59735), p-EGFR (#11862), EGFR (#4267), Ki67 (#12202) and HRP-conjugated anti-rabbit IgG antibody (#7074) were purchased from Cell Signaling Technology (Boston, MA, United States). Cell Counting Kit-8 (CCK-8), Calcein-AM/PI double staining kit, Annexin V-FITC/PI Kit and iron assay kit were obtained from Beyotime Biological Technology Co. Ltd. (Shanghai, China). BODIPY™ 581/591 C11 was purchased from ThermoFisher Scientific (Waltham, MA). Other regents were purchased from Sigma-Aldrich (St. Louis, MO, USA).

### Cell lines and cell culture

The Human non-small cell lung cancer cells PC9, HCC827, NCI-H1975, NCI-H1299, Murine macrophage RAW246.7 cells and Human microvascular endothelial cells (HMEC-1) were from American Type Culture Collection (ATCC, Manassas, VA, USA). The PC9 cells resistant to osimertinib (PC9-OR), NCI-H1975 cells resistant to osimertinib (NCI-H1975-OR) and HCC827 resistant to osimertinib (HCC827-OR) were established by our laboratory. The cells were cultured with RPMI 1640 medium containing 10% FBS, 1% penicillin/streptomycin solution. All the cells were maintained in an incubator containing 5% CO_2_ at 37 °C.

### Cell proliferation and colony formation assay

The cells viability was detected with CCK-8 kit. The cells were seeded at 96-well plates and treated with or without HBT for 48 h. After removing the cultured medium, 10-μL CCK-8 agents were added to the cells and cultured for another 2 h. And then, the absorbance was measured using a SynergyMx Multi-Mode Microplate Reader (Biotek, Winooski, VT) at 450 nm.

As for the colony formation assay, 1000 cells/well were seeded in 6-well plates and treated with different concentration of HBT for 72 h. And the cells were cultured with fresh medium for 2 weeks and stained with crystal violet solution. The colony number was observed and photographed using an EVOS XL Core (Thermo Fisher Scientific).

### Calcein-AM/propidium Iodide (PI) staining assay

The number of living and dead cells was detected with Calcein-AM/PI assay. After treating with HBT for 48 h, the cells were stained with 2 µM Calcein-AM regent and 4.5 µM PI regent for 30 min at 37 °C. And viable cells and dead cells were observed and photographed with confocal microscope.

### Lipid peroxidation assay

To measure the effect of HBT on ROS, PC9-OR cells were seed in 6-well and cultured for 24 h. Following pretreatment with HBT for 24 h, the cells were stained with 2 μmol/L DCFH-DA (Invitrogen, USA) for 30 min and then and subjected to confocal microscope to observe the fluorescence intensity. To assess the effect of HBT on lipid peroxidation, PC9-OR cells were treated with various concentrations of HBT for 24 h, then stained with 10 μM C11-BODIPY 581/591 for 20 min, followed by flow cytometry detection.

### Annexin V-FITC/PI assay

The apoptotic cells were detected with AnnexinV-FITC/PI kit according to the manufacturer's instructions. The PC9-OR cells (3 × 10^5^ cells/well) were seeded in 6-well plates for 24 h, following with indicated concentration of HBT for 48 h. The cells were harvested and stained with Annexin V-FITC and PI solution, subsequently subjected to flow cytometry for fluorescence intensity estimation.

### Measurement of ferrous iron

Fe^2+^ was detected using iron assay kit as previous study described [15]. PC9-OR cells were treated with indicated concentration of HBT for 24 h. And then, the cells were harvested and applied for flow cytometry to analyze the fluorescence intensity.

### Transmission electron microscopy (TEM)

Following treating with HBT for 48 h, the PC9-OR cells were fixed in 3% phosphate-glutaraldehyde for 2 h and post-fixed using 1% osmium tetroxide. After that, the cells were dehydrated with a graded ethanol series, embedded in Epon Resin 618, and then cut into ultrathin sections (60–80 nm). After staining with 2% uranyl acetate saturated alcohol solution and lead citrate, the section was observed and photographed with a TEM (HT7700 EXALENS, HITACHI, Japan).

### Western blotting assay

The PC9-OR cells with indicated treatment were lysed with RIPA buffer containing protease inhibitor cocktail. After measuring protein concentration, equal amount of proteins (30 μg per sample) were separated in SDS-PAGE gels and transferred to a PVDF membrane. And the membrane was blocking with 5% BSA, and incubated with primary antibodies against p-EGFR, EGFR, GPX4, SLC7A11, SCD1, NCOA4, NRF2 and GAPDH overnight at 4 °C. And then, the membrane was incubated with secondary antibody for 1 h at room temperature. The blots were visualized with an ECL detection reagent.

### RNA-seq analysis

The RNA-seq analysis was performed with Seqhealth Technology Co., Ltd. (Wuhan, China). The PC9-OR cells with or without HBT treatment were collected and subjected for RNA extraction. After qualitative and quantitative tests, the RNA was applied for library preparation, and the library products were further sequenced with the Illumina NovaSeq 6000 sequencing platform. Differentially expressed genes (DEGs) between vehicle group and BT group were identified using the limma package with an adjusted screened using thresholds of | log2 (fold change)|> 2 and p-value < 0.05. And the heatmap and volcano plots of DEGs were generated using the ggplot2 plotting module (version 3.3.2).

### RNA extraction and quantitative real-time polymerase chain reaction (RT–qPCR)

Total RNA was extracted from the indicated treatment cells using TRIzol and the RNA was used to perform reverse transcription assay to generate cDNA. And RT-qPCR assay was conducted using a SYBR Green PCR kit (Takara) as our previous study described [16]. The GAPDH was set as endogenous control. The sequences of the primers were as follow: SCD1 forward:5′ TGTGGTGAAGTTGATGTGCCAGC 3′ and reverse 5′ CCTGGTTTCACTTGGAGCTGTG 3', GAPDH forward 5′ TGAGCGATGTGGCTCGGCT3′ and reverse 5′CTCTCTGCTCCTCCTGTTCGAC3′.

### Construction of xenograft tumor model

Four-week-old male BALB/c nude mice were purchased from Hua Fukang Experimental Animal Center (Beijing, China) and housed under specific pathogen-free conditions and maintained on a 12 h light–dark rhythm in the animal laboratory of Guangzhou medical university. The mice were subcutaneously injected with PC9-OR cells with 1 × 10^7^ cells per mice. When the tumor volume reached about 100 mm^3^, the mice were intraperitoneal injected with normal saline, 5 mg/kg osimertinib group, 2.5 mg/kg HBT group and 1.25 mg/kg HBT group every two days for totally 18 days. At the end of the experiment, the mice were humanely anesthetized by inhalation of isoflurane and then sacrificed. The tumors were removed, weighted and photographed. And then, the mice were fixed with 4% polyformaldehyde for further pathological examination.

### Hematoxylin and eosin (H&E) staining and immunohistochemistry (IHC) assay

The fixed tumor tissues were embed using paraffin and sectioned into 5 μm slides. The sections were deparaffinized and rehydrated in a descending series of alcohol. And then, the sections were immersed in a solution of hematoxylin for 3 min and stained with eosin for 5 min subsequently. As for the IHC assay, the sections were subjected for standard protocol for deparaffinization and rehydration. And then, the sections were stained with primary antibody against Ki67, SCD1 and GPX4 overnight at 4 °C. After that, the sections were incubated with HRP labeled goat anti-mouse/rabbit secondary antibody at room temperature for 1 h, following with DAB chromogen solution. Finally, the sections were counterstained with hematoxylin. The microscopic images were acquired using a microscope.

As for the IHC assay of Human lung cancer tissues, 16 patients diagnosed with NSCLC were include in this study. Ethics approval was obtained from the Institutional Review Board (IRB) of the Sixth Affiliated Hospital of Guangzhou Medical University (approval number: IRB‑2021‑025). The written informed consent according to guidelines of the Declaration of Helsinki have been obtained from all the patients. The sections were blocked using 5% BSA and incubated with primary antibody against SCD1, followed by HRP labeled goat anti-rabbit secondary antibody at room temperature for 1 h and DAB chromogenic reaction. The integrated optical density (IOD) values of SCD1 were analyzed using Image‑Pro Plus 6 software. And the clinical baseline data of the 16 NSCLC patients were listed in supporting Table 1.

### SCD1 vector and siRNA transfection

For overexpression of SCD1, the gene was cloned into pcDNA3.1-3 vector to generate the recombinant pcDNA3.1-3-SCD1 vector. The PC9-OR cells were transfected with pcDNA3.1-3-SCD1 vector using Lipofectamine 3000 Transfection Reagent following with the protocol of the manufacturer. As for the siRNA transfection, the PC9-OR cells were transduced with SCD1 siRNA/NC siRNA using Lipofectamine 3000 reagent.

After 48-h transfection, the cells were collected and subjected for Western blotting assay to validated the transfection efficacy. The oligonucleotides targeting SCD1 were listed as follow: CTACGGCTCTTTCTGATCA.

### Molecular docking

The automated docking simulation was conducted by the AutoDock Tools (ADT, ver.1.5.7). The 3D structure of SCD1 complexed with its substrate stearoyl-CoA (PDB ID: 4zyo) was from the Protein Data Bank (http://www.rcsb.org). The 3D structure of HBT was constructed through ChemBio3D Ultra 20.0 software with MM2 force field. Initially, the protein structure was prepared for docking using the PyMOL program, which involved removing water molecules and adding hydrogen atoms. Subsequently, the grid map was generated by the AutoGrid program with a grid box size of 50 × 58 68 and a default spacing of 0.375 Å. The grid center coordinates for SCD1 were set to X = 20.108, Y = 68.147, and Z = 45.093. Molecular docking was executed on the AutoDock 4.2 platform with default parameters. The pose with the lowest binding energy was then selected for further analysis using PyMOL software.

For the molecular dynamic stimulation, he top-ranked docking pose of HBT bound to hSCD1 (PDB ID: 4ZYO) was subjected to 100 ns all-atom molecular dynamics simulation using GROMACS 2023.2. The protein was modeled with the AMBER99SB force field, and ligand parameters were generated by Sobtop. The complex was solvated in a cubic box of TIP3P water with a minimum solute–box distance of 10 Å, neutralized with Na⁺/Cl⁻ ions, and energy-minimized via steepest descent. Equilibration involved 100 ps NVT at 310.15 K (V‑rescale) and 100 ps NPT at 1 bar (Parrinello–Rahman), followed by a 100 ns NPT production run (2 fs step). Hydrogen bonds were constrained with LINCS, long-range electrostatics treated with PME, and coordinates saved every 10 ps. Protein/ligand RMSD, radius of gyration, and solvent-accessible surface area were analyzed. Snapshots at 0, 50, and 100 ns were superimposed onto the initial pose to assess binding mode preservation. Visualizations were prepared with PyMOL.

### Biacore binding assay

The surface plasmon resonance (SPR) analysis was conducted using a BIAcore X100 instrument (GE-Healthcare, Milwaukee, WI, USA) with research-grade CM4 carboxyl-methyl-dextran-coated sensorchips (GE-Healthcare) with a using research-grade CM5 carboxyl-methyl-dextran-coated sensorchips (GE-Healthcare). The SCD1 protein was resuspended at 20 μg/mL and then injected onto the CM5 Chip (GE, USA) for immobilization. Several concentrations of HBT dissolved in running buffer were flown over the chip to produce response signals. The kinetics and affinities between HBT and SCD1 were calculated by the Biacore Insight Evolution software, and the results were determined as the binding affinity (KD).

### Protein stability assay of SCD1

The PC9-OR cells were pre-treated with CHX for 6 h, and then the cells were treated with or without HBT for 0 h, 2 h, 4 h, 6 h, 12 h and 24 h. After that, the cells were collected and applied for western blotting assay to detect SCD1 expression.

### Cellular thermal shift assay (CETSA)

The CETSA was conducted to evaluate the effect of HBT on SCD1. The PC9-OR cells were collected and lysed using RIPA buffer containing protein inhibitor. The supernatants were collected through centrifuging at 12,000*g* for 15 min at 4 °C. And equal proteins were treated with or without HBT for 30 min at 25 °C. Subsequently, the protein of each group was divided into 6 equal parts and heated at 41 °C, 44 °C, 47 °C, 50 °C, 53 °C, 56 °C for 3 min each. After that, the protein was subjected for Western blotting assay.

### Statistical analysis

GraphPad Prism 8.0 (GraphPad Software Inc., San Diego, CA, USA) was used for statistical analysis. Normality was assessed by Shapiro–Wilk test, and variance homogeneity by Levene’s or Bartlett’s test. For two-group comparisons, an unpaired two-tailed t-test was used. For three or more independent groups, one‑way ANOVA followed by Tukey’s post‑hoc test (parametric) was used. For repeated-measures data (e.g., tumor volume), two-way repeated-measures ANOVA with Greenhouse–Geisser correction (when Mauchly’s test for sphericity was violated) was employed to evaluate the effects of group, time, and their interaction. Survival curves were generated by the Kaplan–Meier method and compared by the log‑rank test. “n” represents biological replicates for in vitro experiments (technical replicates averaged) or individual mice for in vivo experiments. When p value < 0.05, it was considered as statistically significant.

## Results

### HBT inhibits the proliferation of osimertinib-resistant NSCLC cells

**Fig. 1:**
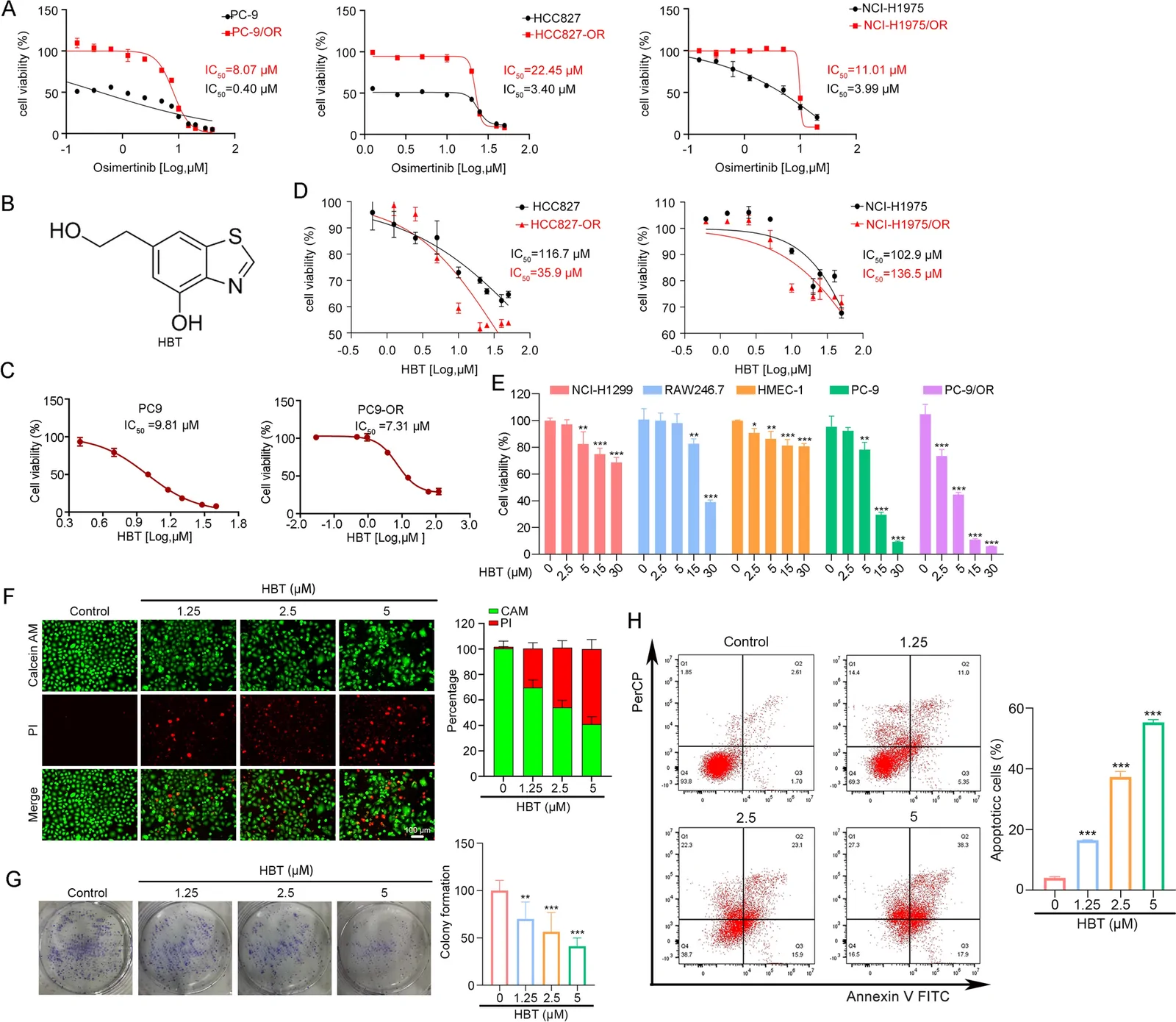
HBT inhibits PC9-OR cell proliferation. **A** The IC_50_ of osimertinib on PC9, PC9/OR, HCC827, HCC827/OR, NCI-H1975 and NCI-H1975/OR cells. **B** The chemical structure of HBT. **C** The IC_50_ of HBT on PC9 cells and PC9-OR cells. The cells were treated with various concentrations of HBT for 48 h, and the cell viabilities were detected with CCK-8 assay. **D** The IC_50_ of HBT on HCC827, HCC827/OR, NCI-H1975 and NCI-H1975/OR cells. **E** The effect of HBT on the cell proliferation of NCI-H1299, RAW246.7 and HMEC-1 cells. **F**,** G** HBT inhibited the PC9-OR cell proliferation indicated by Calcein-AM/PI staining assay and colony formation assay. The representative images and quantitative data were presented. **H** HBT induced the apoptosis of PC9-OR cells detected by Annexin V-FITC/PI assay. After treating with various concentration of HBT for 48 h, the PC9-OR cells were collected and incubated with Annexin V-FITC and PI reagent, and then subject for flow cytometry. All the data were presented as mean ± SD. n = 3. ^*^P < 0.05, ^**^P < 0.01, ^***^P < 0.001 compared with the Vehicle group

We generated osimertinib-resistant sublines of PC9 (PC9-OR), HCC827 (HCC827-OR), and NCI-H1975 (NCI-H1975-OR) and evaluated their sensitivity to osimertinib using the CCK-8 assay. The results showed that these sublines are less sensitivity to osimertinib than their parent cells, with fold changes in IC₅₀ values of 20.18, 6.60, and 2.76, respectively (Fig. 1A and Supporting Fig. 1). We evaluated the effect of HBT (Fig. 1B) on the cell viability of PC9-OR cells, NCI-H1975-OR cells and HCC827-OR cells. The results revealed that HBT significantly suppressed the proliferation of PC9-OR and its parental cells with PC9 cells with an IC_50_ of 7.31 μM and 9.81 μM (Fig. 1C). However, HBT showed weaker effect on the proliferation of NCI-H1975-OR/NCI-H1975 and HCC827-OR/HCC827 cells (Fig. 1D). Thus, we selected PC9-OR cells for further evaluation. We also found that HBT showed better antiproliferative activity against PC9-OR cells compared to PC9 cells at concentrations of 2.5, 5, 15, and 30 μM, as well as compared to several NSCLC or normal cells, including NCI-H1299, RAW 246.7 and HMEC-1 cells (Fig. 1E). Calcein-AM/PI assay showed that HBT induced PC9-OR cell death in a dose-dependent manner (Fig. 1F). Colony formation assay also revealed that HBT treatment obviously reduced the colony number of PC9-OR cells. The colony number in 5 μM HBT group is only about 40% of control group (Fig. 1G). Similar results were observed in Annexin V-PI assay, HBT treatment leading to obvious PC9-OR cell necrosis or apoptosis in a dose dependent manner (Fig. 1H). All these results demonstrated that HBT significantly suppressed PC9-OR cell proliferation.

### HBT triggers ferroptosis in osimertinib resistance cells

**Fig. 2:**
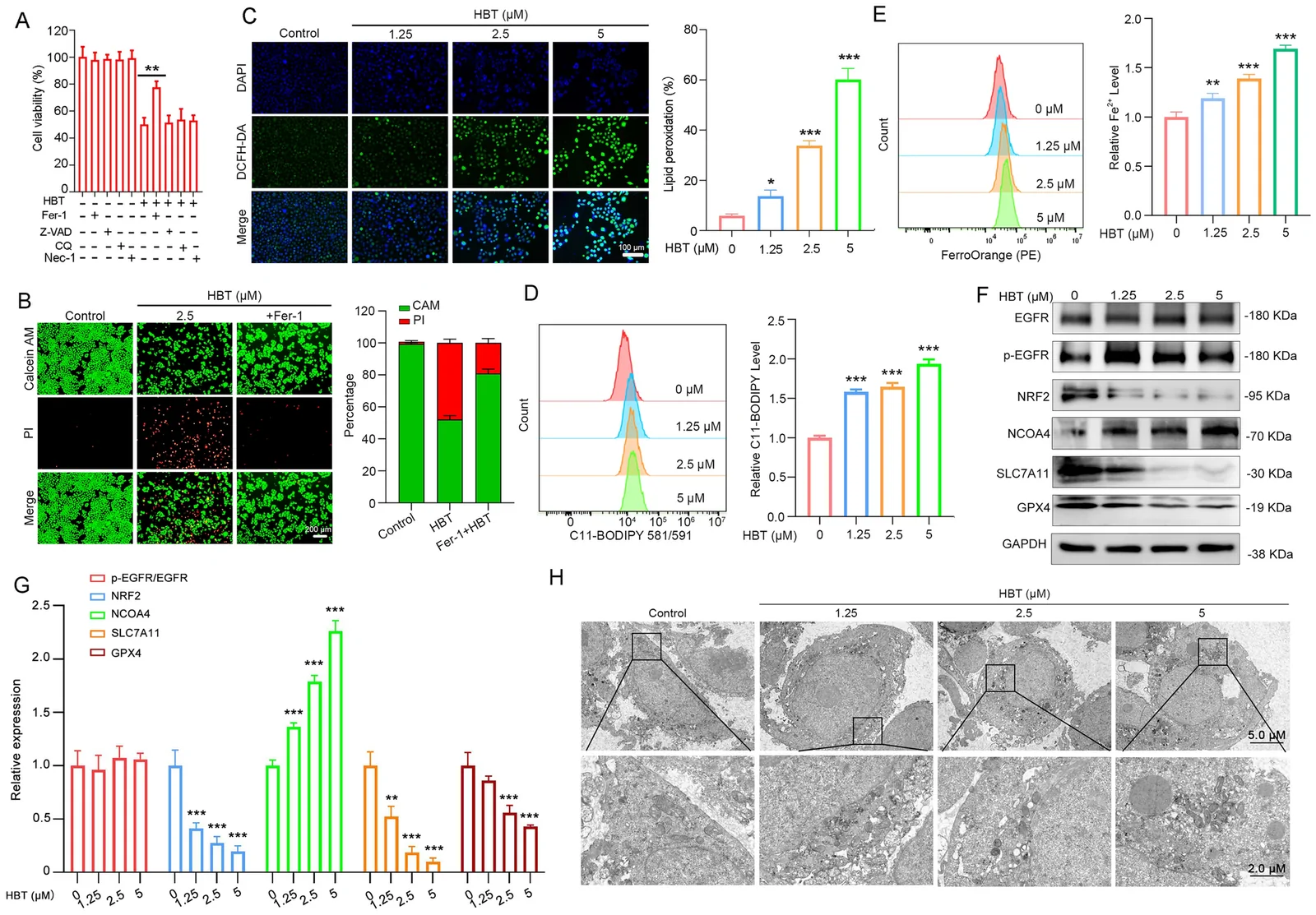
HBT triggers PC9-OR cells ferroptosis. **A** Fer-1 treatment attenuated 2.5 μΜ HBT-caused inhibition effect on PC9-OR cell proliferation. The PC9-OR cells were co-treated with apoptosis inhibitor Z-VAD-FMK, ferroptosis inhibitor Fer-1, autophagy inhibitor CQ or necroptosis inhibitor Nec-1 and HBT for 48 h, and then subjected for CCK-8 assay. **B** Fer-1 treatment weaken HBT-mediated PC9-OR cells death detected by Calcein-AM/PI staining assay. **C** HBT treatment induced intracellular ROS accumulation. **D** HBT treatment increased intracellular lipid peroxidation, as indicated by C11-BODIPY 581/591 staining assay. **E** HBT treatment increased intracellular Fe^2+^ level. The PC9-OR cells were treated with various concentration of HBT for 12 h, and the Fe^2+^ level were detected by flow cytometry. **F**, **G** HBT induced PC9-OR cells ferroptosis indicated by Western blotting assay. **H** HBT treatment induced mitochondrial morphology change. PC9-OR cells were treated with or without HBT for 24 h. And the subcellular organelle structure was observed using TME. All the data were presented as mean ± SD. n = 3. ^**^P < 0.01, ^***^P < 0.001 compared with the Vehicle group

Next, we further explore how HBT induced PC9-OR cell death. Several cell death inhibitors, including Z-VAD-FMK (an apoptosis inhibitor), Fer-1 (a ferroptosis inhibitor), CQ (an autophagy inhibitor) and Nec-1 (a necroptosis inhibitor) were combined with HBT. The results revealed that Z-VAD-FMK, CQ and Nec-1 had mimic effect on HBT-induced PC9-OR cell death. Whereas, Fer-1 treatment obviously restored HBT-induced PC9-OR cell death (Fig. 2A). Calcein-AM/PI staining assay also confirmed the result. HBT obviously induced PC9-OR cells death. Whereas, Fer-1 treatment significantly blunted HBT mediated inhibition effect on PC9-OR cell death (Fig. 2B). These results suggest that HBT may induce PC9-OR cells ferroptosis. To further validate whether HBT induced PC9-OR cell ferroptosis, several experiments were performed to evaluate the effect of HBT on biochemical processes of ferroptosis. As expected, DCFH-DA staining revealed that HBT treatment significantly increased ROS levels in PC9-OR cells (Fig. 2C), and C11-BODIPY 581/591 staining confirmed a concurrent rise in intracellular lipid peroxidation (Fig. 2D). Fe^2+^ level detection also showed that HBT treatment leading to increased Fe^2+^ level of PC9-OR cells (Fig. 2E). Western blotting showed that HBT did not substantially reduce p‑EGFR or total EGFR levels, suggesting that HBT mediated inhibition effect on PC9-OR cells may not be mediated primarily through direct EGFR inhibition. Furthermore, HBT treatment decreased NRF2, SLC7A11 and GPX4 expression in a dose dependent manner, along with the upregulation of NCOA4 (Fig. 2F and G). TME assay was conducted to observe the effect of HBT on mitochondrial morphology of PC9-OR cells. We found that HBT treatment decreased mitochondrial volume and increased membrane density. Even more, there was almost no mitochondrial ridges were observed in 5 μM HBT group (Fig. 2H). These findings suggest that HBT might promoted PC9-OR cells death by inducing ferroptosis.

### SCD1 is associates with HBT-induced PC9-OR cells ferroptosis

**Fig. 3:**
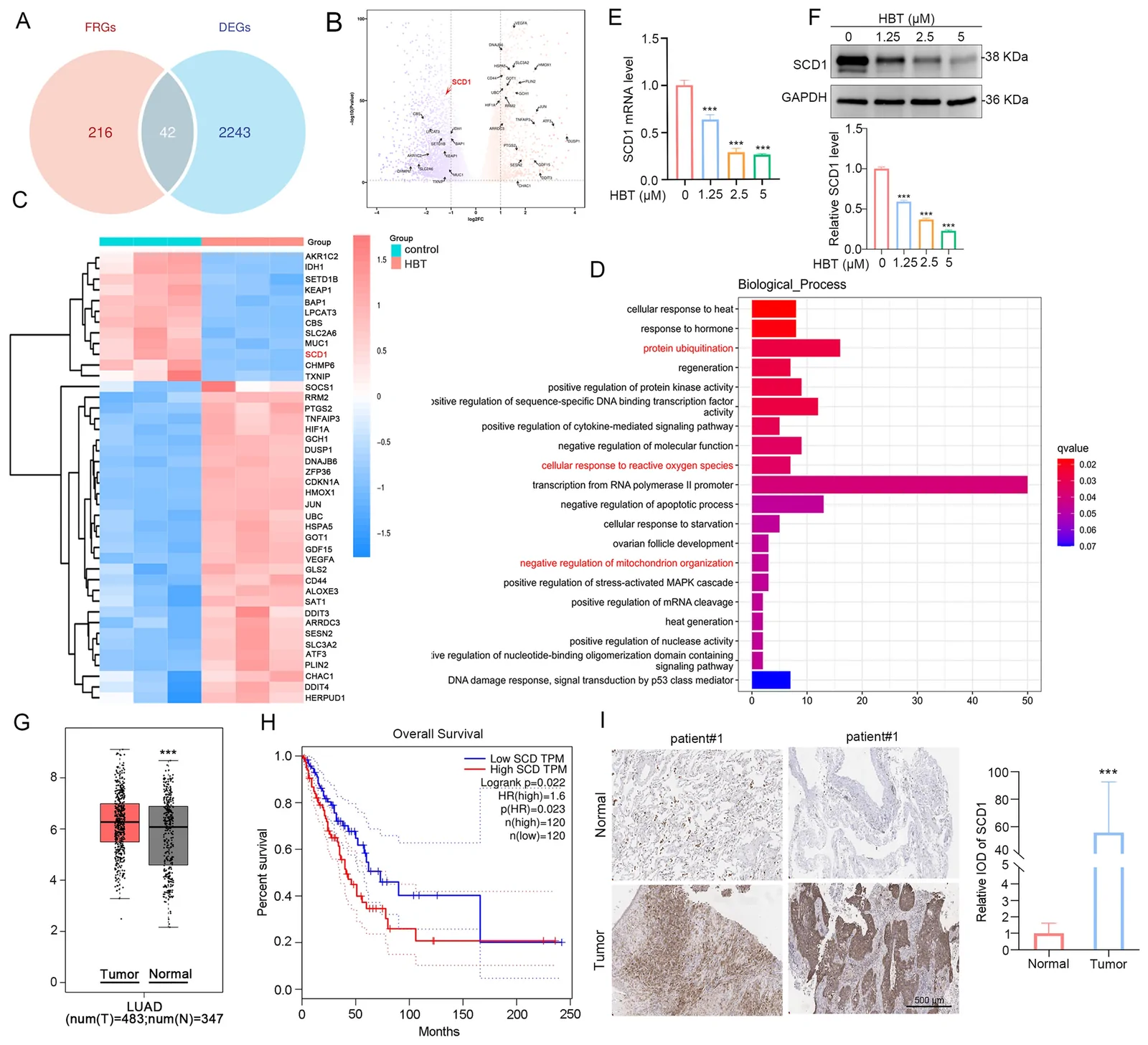
SCD1 is involveed in HBT-induced ferroptosis. **A** Venn diagram of the overlapping different expressed genes and ferroptosis related genes. **B** The volcano plot shows different expression genes. **C** Heatmap represents 42 differentially expressed genes. **D** Gene ontology analysis based on RNA sequences. **E**, **F** RT-qPCR assay and Western blotting assay confirmed that HBT decreased SCD1 expression. The results of RT-qPCR assay and Western blotting assay were presented in **E** and **F**. **G** SCD1 expression in lung adenocarcinoma tissues and normal tissues indicated by TCGA data. **H** The patient survival of NSCLC patients with different SCD1 expression. **I** IHC assay indicate SCD1 expression in NSCLC tissues and adjacent human tissues. n = 8. All the data were presented as mean ± SD. n = 3. ^**^P < 0.01, ^***^P < 0.001 compared with the vehicle group

To further explore how HBT induce ferroptosis, we analyze the RNA profiles of PC9-OR cells with or without HBT treatment. The results showed that there were 2243 different genes between vehicle cells and HBT-treated cells. Among them, there are 42 genes overlapped with ferroptosis related gene based on Venn diagram analyze (Fig. 3A and B). And SCD1 is one of the ferroptosis regulators that showed a significant decrease (Fig. 3C). Gene enrichment analysis indicated that the differentially expressed genes upon HBT treatment were associated with several pathways, including those related to ferroptosis, the endoplasmic reticulum, and mitochondria (Fig. 3D). These findings are consistent with the hypothesis that HBT triggers ferroptosis in PC9-OR cells. RT-qPCR assay and Western blotting assay demonstrated that HBT obviously reduced SCD1 expression in a dose dependent manner (Fig. 3E and F). ITGA data analyze showed SCD1 expression in lung adenocarcinoma tissues obviously increased when compared with the adjacent tissues (Fig. 3G). And lung adenocarcinoma patients with high SCD1 level have poorer prognosis than that with low SCD1 expression (Fig. 3H). IHC assay also revealed that SCD1 level was significantly up-regulated in NSCLC tissue when compared with the adjacent tissues (Fig. 3I). Thus, SCD1 was identified for further investigation.

### HBT induces ferroptosis partly dependent on SCD1

**Fig. 4:**
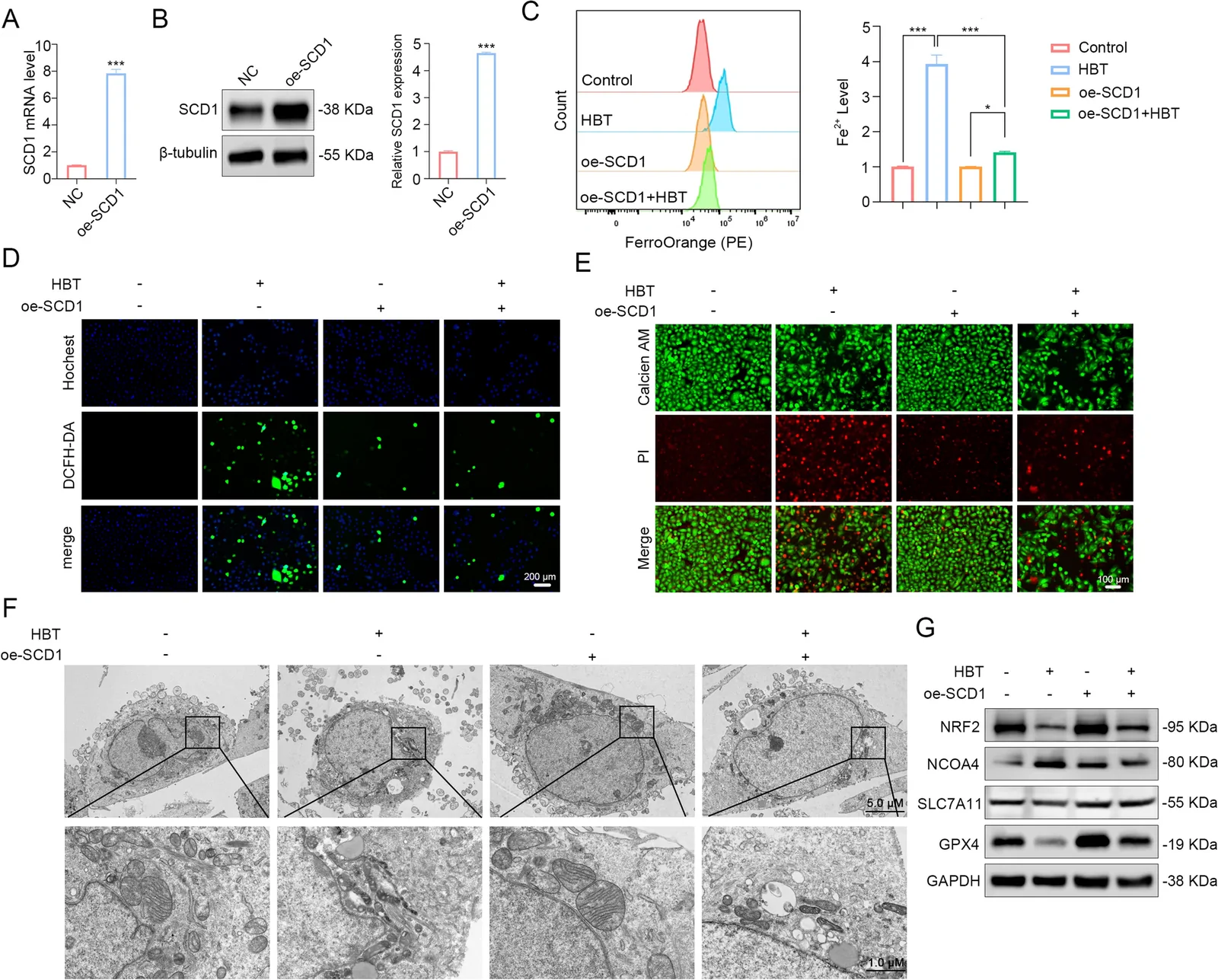
SCD1 overexpression attenuates HBT-induced ferroptosis on PC9-OR cells. **A**, **B** SCD1 was overexpressed in PC9-OR cells. The cells were transfected with NC vector or SCD1 vector, and the transfection efficacy was confirmed using RT-qPCR assay (**A**) and Western blotting assay (**B**). **C** SCD1 overexpression attenuated HBT induced iron overload. **D** SCD1 overexpression weaken HBT-induced intraocular ROS level. **E** SCD1 overexpression broken HBT mediated inhibition effect on PC9-OR cells. **F** SCD1 overexpression relieved HBT-induced mitochondrial morphological changes. **G** SCD1 overexpression attenuated HBT mediated inhibition effect on ferroptosis related protein. All the data were presented as mean ± SD. n = 3. ^**^P < 0.01, ^***^P < 0.001

To further evaluate whether SCD1 is necessary for HBT-induced ferroptosis, PC9-OR cells were transfected with SCD1 vector. RT-qPCR assay and Western blotting assay demonstrated that SCD1 was overexpressed in PC9-OR cells (Fig. 4A and B). HBT treatment obviously increased Fe^2+^ level of PC9-OR cells, whereas SCD1 overexpression significantly attenuated iron overload (Fig. 4C). DCFH-DA probe detection staining also confirmed that SCD1 overexpression obviously attenuated HBT-induced intracellular ROS accumulation (Fig. 4D). Calcein-AM/PI staining revealed that HBT obviously induced PC9-OR cells death, whereas SCD1 overexpression revered HBT-induced PC9-OR cells death (Fig. 4E). Moreover, HBT induced mitochondria shrinkages and folds was obviously blunted with SCD 1 overexpression (Fig. 4F). Western blotting assay also showed that HBT treatment decreased GPX4, SLC7A11 and NRF2 expression, whereas SCD1 overexpression obviously mitigated HBT-induced GPX4 and NRF2 downregulation (Fig. 4G).

**Fig. 5:**
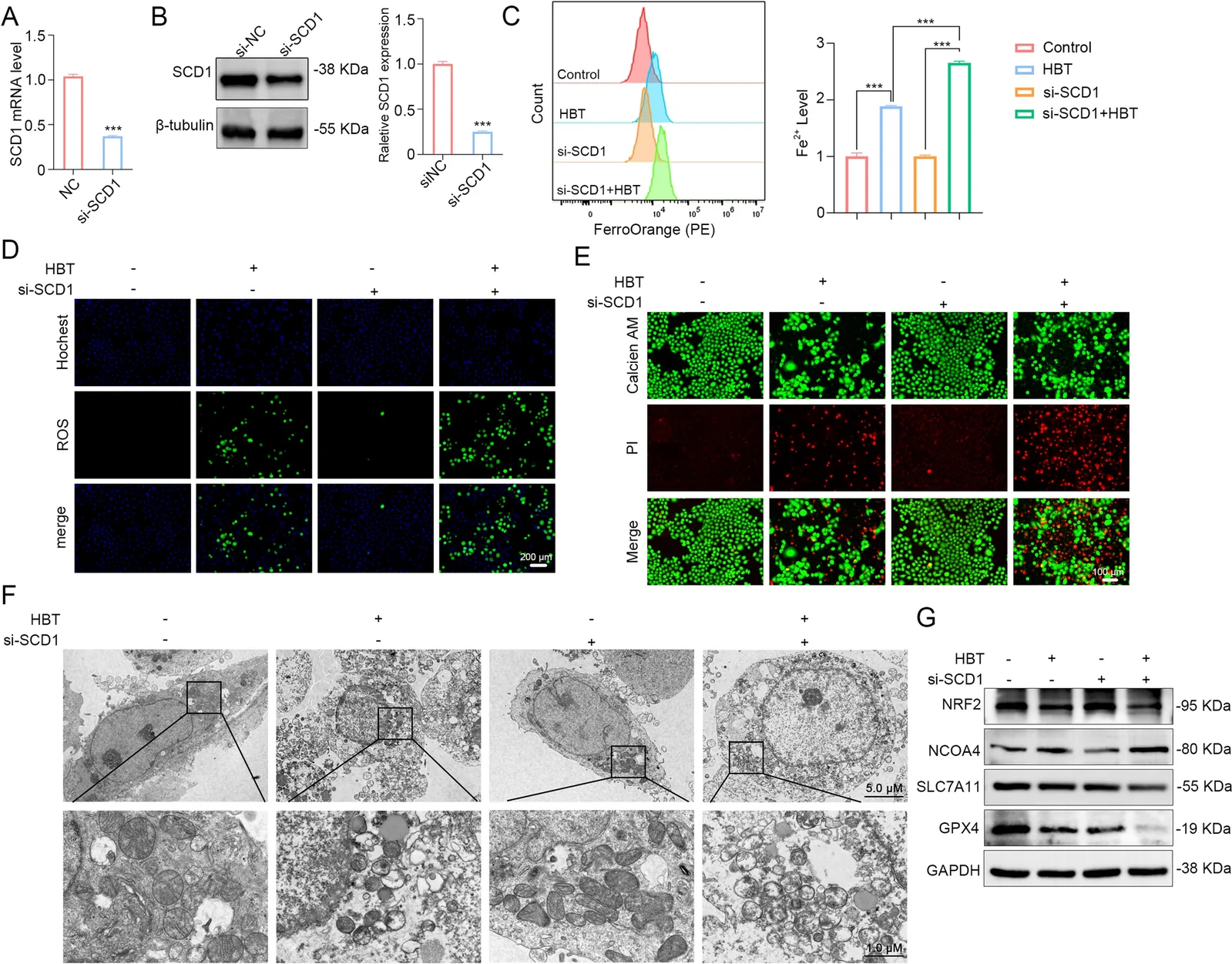
SCD1 knockdown enhances HBT mediated ferroptosis on PC9-OR cells. **A**, **B** SCD1 was silenced in PC9-OR cells. PC9-OR cells were transfected with or without siRNA SCD1. RT-qPCR assay (**A**) and Western blotting assay (**B**) was performed to validate the transfection efficacy. **C** SCD1 knockdown accelerated HBT mediated Fe^2+^ level increase. **D** SCD1 silence enhanced HBT-induced cell death. PC9-OR cells transfected with siRNA SCD1 and then treated with HBT for 48 h. The cells were stained with Calcein-AM/PI solution and overserved. **E** SCD1 knockdown increased HBT-induced ROS accumulation. **F** SCD1 knockdown accelerated HBT-induced mitochondrial membrane wrinkle. **G** SCD1 silence enhanced HBT-induced inhibition effect on several critical ferroptosis related protein. All the data were presented as mean ± SD. n = 3. ^**^P < 0.01, ^***^P < 0.001

SCD1 was also knockdown in PC9-OR cells to further validate its role in HBT-induced ferroptosis. RT-qPCR assay and Western blotting assay verified SCD1 was successfully silenced in PC9-OR cells (Fig. 5A and B). Fe^2+^ level detection assay showed that SCD1 siRNA transfection significantly enhanced HBT-mediated iron overload of PC9-OR cells (Fig. 5C). SCD1 knockdown also effectively aggravated HBT induced intrasellar lipid peroxidation (Fig. 5D). Calcein-AM/PI staining result confirmed that HBT-induced PC9-OR cell death was obviously enhanced by siRNA SCD1 transfection (Fig. 5E). Similar results were observed in TME assay and Western blotting assay. SCD1 silences markedly enhanced HBT mediated mitochondrial membrane wrinkling (Fig. 5F). Western blotting assay revealed that HBT combine with SCD1 siRNA obviously reduced NRF2, SLC7A11 and GPX4 expression when compared with HBT treatment group (Fig. 5G). All these data suggest that HBT trigger ferroptosis partly in a SCD1 dependent manner.

### HBT is a novel SCD1 inhibitor

**Fig. 6:**
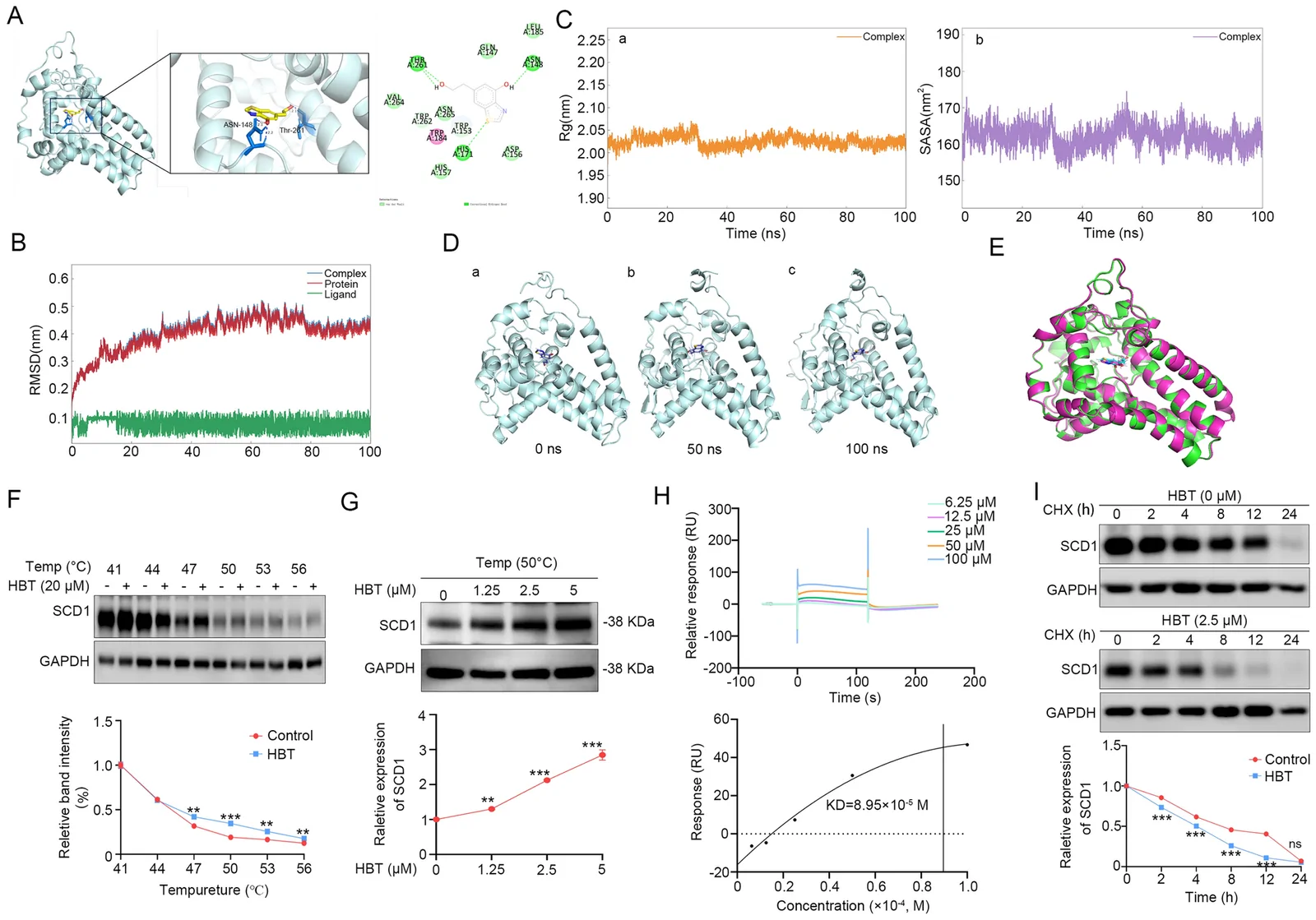
HBT decreases SCD1 expression by promoting its degradation. **A** Hydrogen bonds between HBT and the residues Asn-148 and Thr-261 of the stearoyl-CoA-binding site. The interactions of HBT to the amino acid residues in the stearoyl-CoA-binding site of SCD1 were shown. **B** Time evolution of the RMSD values of the hSCD1-HBT complex, hSCD1 protein backbone, and HBT ligand during the 100 ns MD simulation. **C** Global conformational stability of the HBT–hSCD1 complex during the 100 ns MD simulation. Radius of gyration (Rg) and Solvent-accessible surface area (SASA) of hSCD1 as a function of simulation time were displayed. **D** Representative snapshots of the HBT-hSCD1 complex extracted at 0 ns (left), 50 ns (middle), and 100 ns (right) of the MD trajectory. **E** Superposition of the equilibrated MD structure (100 ns) and the initial docking pose of the HBT-hSCD1 complex. The initial docking pose is shown in cyan and the 100 ns MD structure in magenta. **F** CETSA assay detect the thermostability between HBT and SCD1. **G** HBT decreased the thermostability of SCD1 in a dose dependent manner. **H** Biacore X100 detected the kinetics/affinity of HBT and SCD1. **I** HBT inhibited the SCD1 protein stability. PC9-OR cells were treated with HBT for 24 h, and then treated with CHX (10 μM) for 0 h, 2 h,4 h and 6 h. After that, the cells were collected and subjected for Western blotting assay to detect SCD1 expression. All the data were presented as mean ± SD. n = 3. ^**^P < 0.01, ^***^P < 0.001

To investigate the interaction between HBT and SCD1, we conducted a molecular docking analysis. The results showed that HBT forms hydrogen bonds with the residues Asn-148 and Thr-261 of the stearoyl-CoA-binding site. And the binding energy between HBT and SCD1 is − 6.26 kcal/mol (Fig. 6A). Further a 100 ns molecular dynamics simulation showed hSCD1 backbone RMSD plateaued at 0.40–0.50 nm after ~ 40 ns, and ligand RMSD remained below 0.10 nm, indicating stable binding (Fig. 6B). The global structural stability was assessed by radius of gyration (2.00–2.06 nm) and SASA (155–170 nm^2^), both remaining stable with tight distributions and only slight fluctuations. These results indicate that ligand binding did not induce any detectable expansion, unfolding, or loosening of the hSCD1 fold during the simulation (Fig. 6C). Representative snapshots (0, 50, 100 ns) and superposition with the initial docking model confirmed that HBT remained stably anchored in the hydrophobic substrate channel (Fig. 6D and E), supporting a dynamically stable hSCD1 binding mode. Following the outcome of the molecular docking analysis, we performed CETSA to further confirm the interaction of HBT and SCD1, which is based on the principle that ligand binding stabilizes the target protein. As expect, HBT (20 μM) treatment induced obvious thermal shifts of SCD1 at the denaturation temperature range of 41–56 °C (Fig. 6F). We also found that HBT treatment induced thermal shifts of SCD1 in a dose dependent manner at 50 °C, as indicated by the progressively increasing thermal stability of SCD1 at HBT concentrations of 1.25, 2.5 and 5.0 μM (Fig. 6G). Encouraged by the above results, SPR assay was conducted to further validate the interaction between HBT and SCD1. The result revealed that HBT directly interacted with SCD1 with a KD of 8.95 × 10^–5^ M, indicating a moderate binding affinity (Fig. 6H). In addition, protein stability assay showed that HBT treatment leading to SCD1 degradation. The SCD1 protein in vehicle group degraded to an undetectable level at 24 h, whereas SCD1 protein in HBT group was almost undetectable 12 h (Fig. 6I). All these data suggest that HBT directly interacted with SCD1 and then enhanced its degradation.

### HBT suppresses osimertinib resistant tumor growth

**Fig. 7:**
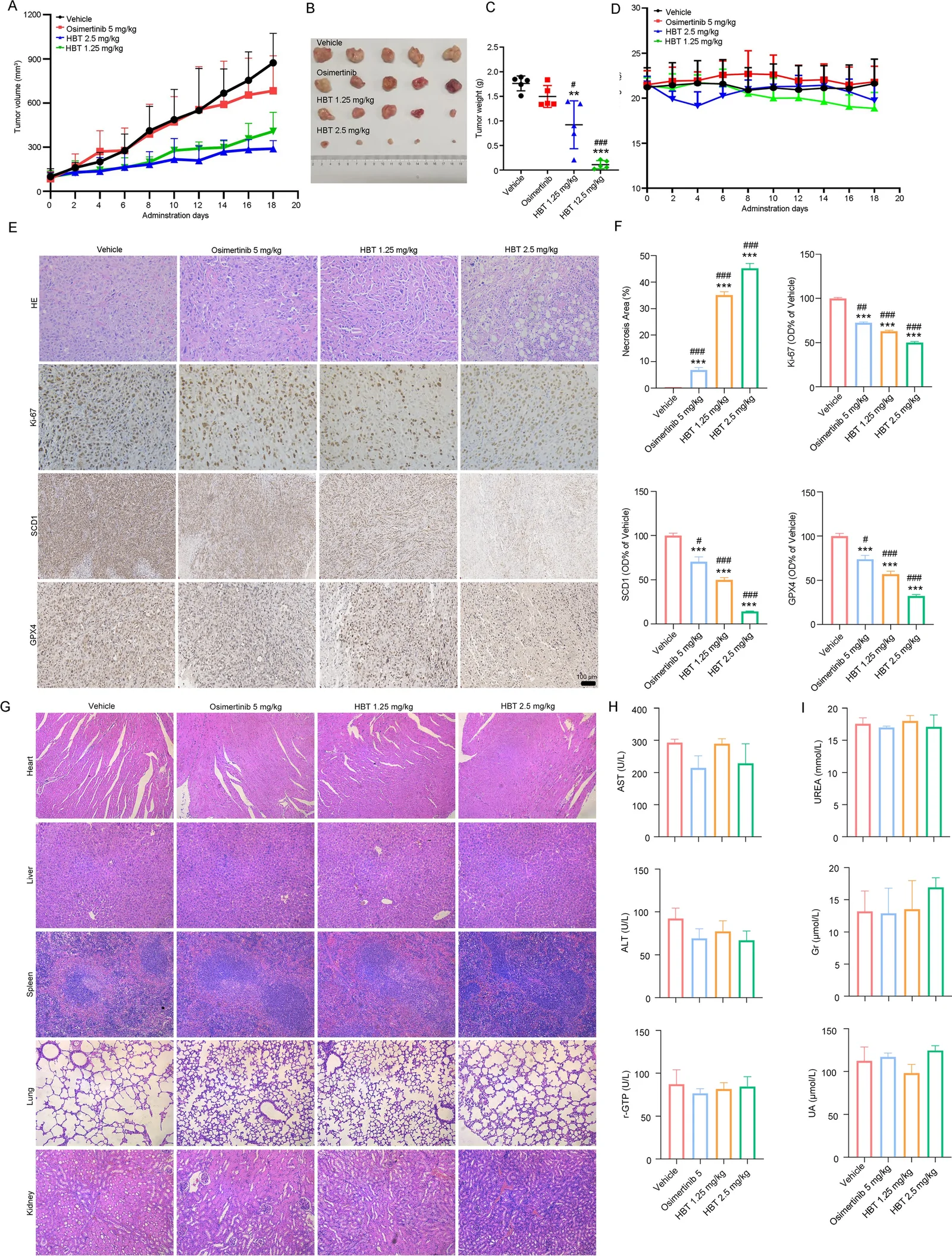
HBT overcome osimertinib resistances of NSCLC. The PC9-OR cells were injected into the right armpits of nude mice to establish xenograft model. The tumor bearing mice were administrated with Vehicle, 20 mg/kg osimertinib, 1.25 HBT or 2.5 mg/kg HBT every two days for totally 18 days. And the tumor volume and mice weight were weighted every two days. The curve of tumor volume (**A**), tumor image (**B**) and tumor weight (**C**) were presented. **D** The effect of HBT on the body weight of mice. **E**, **F** HBT suppressed Ki67, SCD1 and GPX4 expression in mice. IHC assay was conducted to detect the expression of Ki67 and SCD1. **G** The H&E staining of the heart, liver, spleen, lung and kidney of the mice. **H** HBT treatment did not cause hepatotoxicity on the mice. **I** HBT treatment did not induce renal disorder. The representative images and statistical data were displayed. All the data were presented as mean ± SD. n = 5. ^**^P < 0.01, ^***^P < 0.001 vs Vehicle; ^#^*P* < 0.05, ^##^*P* < 0.01, ^###^*P* < 0.001 vs osimertinib

To further validate the effect of HBT in reversing osimertinib resistance, we established a xenograft model using PC9-OR cells. The tumor bearing mice was administrated with 1.25 mg/kg HBT or 2.5 mg/kg HBT. The tumor volume curve showed that HBT administration significantly suppressed the tumor growth when compared with vehicle group or osimertinib group (Fig. 7A). Tumor weight and tumor images also confirmed that HBT showed perfect anti-tumor effect on PC9-OR xenograft (Fig. 7B and C). Moreover, there is no significant change was observed in the mice body weight (Fig. 7D). H&E staining demonstrated that HBT administration leading to loosen tumor tissues and increased necrotic area. Ki67 staining showed that HTB treatment obviously reduced tumor cells proliferation. HBT treatment also reduced GPX4 and SCD1 expression in tumor tissues, indicating that HBT might overcome osimertinib resistance by inducing ferroptosis (Fig. 7E and F). Furthermore, H&E staining revealed no significant histopathological alterations in major organs following HBT administration (Fig. 7G). To systematically evaluate hepatotoxicity and nephrotoxicity, we assessed the levels of key liver and kidney function biomarkers. HBT treatment did not significantly alter the serum levels of liver enzymes, including aspartate aminotransferase (AST), alanine aminotransferase (ALT), and γ-glutamyl transpeptidase (γ-GTP), indicating no obvious hepatotoxicity (Fig. 7H). Similarly, renal function markers such as UREA, creatinine (Cr), and uric acid (UA) remained unaffected by HBT treatment (Fig. 7I). Collectively, these results demonstrate that HBT administration at doses of 1.25 mg/kg and 2.5 mg/kg elicited no significant toxic effects in mice. Combined with its ability to overcome osimertinib resistance, HBT displays a favorable in vivo safety profile.

## Discussion

**Fig. 8:**
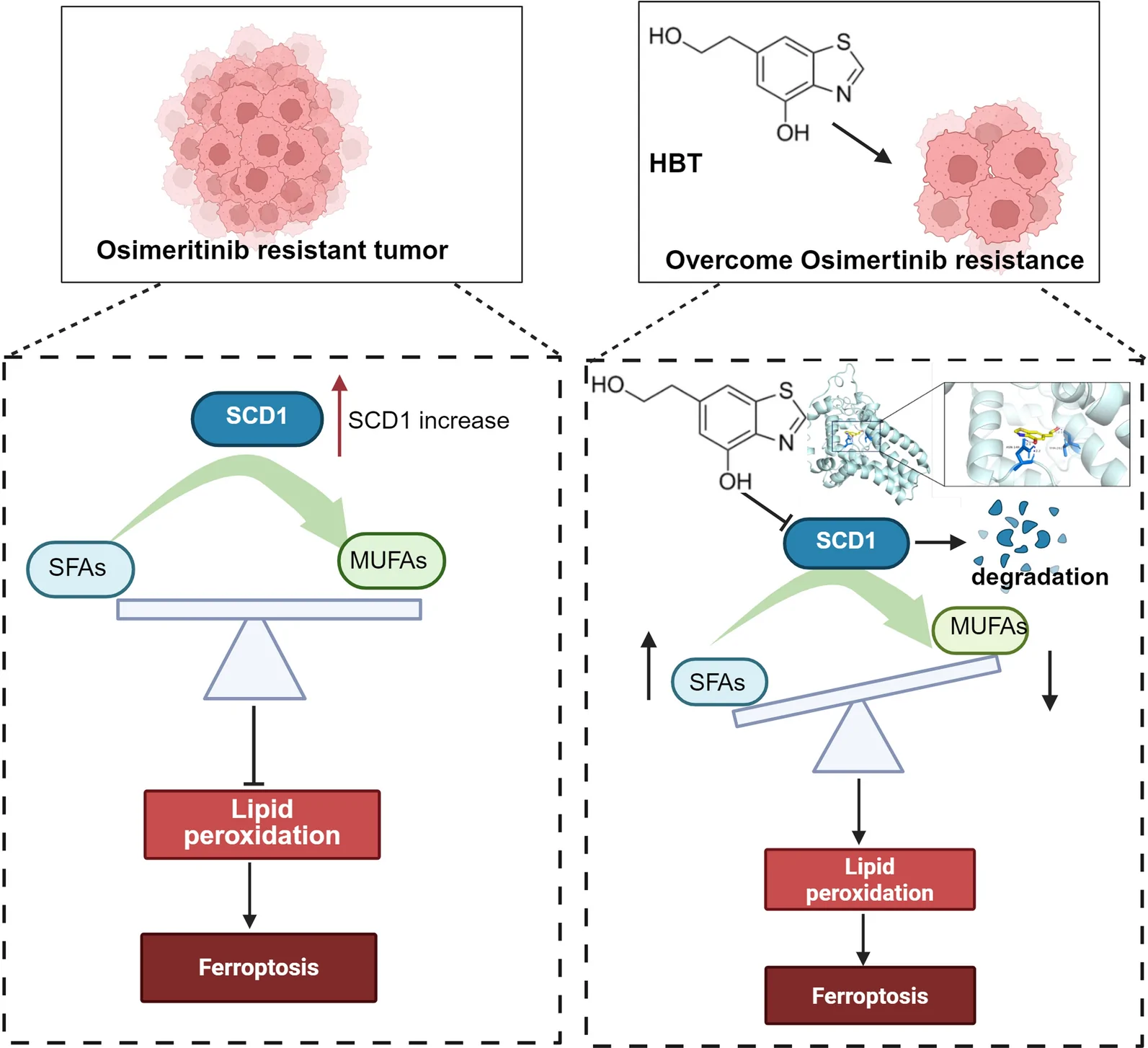
The graphic abstract of the study. HBT overcomes osimertinib resistance by directly binding to SCD1 and enhanced its degradation, leading to lipid peroxidation and ferroptosis

Osimertinib is a third-generation EGFR inhibitor that have been approved by FDA for NSCLC patients with EGFR T790M resistance mutation. And it induces a favorable therapy response and significantly improves many patients’ survival [17]. However, the occurrences of acquired resistance greatly limit its clinical efficacy. The mechanism of osimertinib resistance is heterogeneous, including EGFR-dependent and EGFR-independent manner. Still now there is not effective therapeutic strategy to overcome osimertinib resistance. Thus, identifying novel inhibitor that can reverse osimertinib resistance is meaningful [18, 19]. In the present study, we firstly identified HBT as a SCD1 inhibitor that can reverse osimertinib resistance by inducing ferroptosis. And HBT directly interacted with SCD1 and enhanced its degradation, leading to lipid peroxide and ferroptosis. Moreover, HBT also significantly suppressed tumor growth in PC9-OR xerograph model (Fig. 8). Collectively, our findings indicate that HBT is a potential candidate for counteracting osimertinib resistance.

Emerging studies have showed that ferroptosis contributed to the therapeutic responses of tumors and it was a potential target for chemotherapy resistance [20]. Induction of ferroptosis has been shown to enhance chemosensitivity across multiple cancer types, including colorectal cancer, renal cell carcinoma, and breast cancer [21–23]. Ferroptosis has also been implicated in resistance to EGFR inhibitors. For instance, the ferroptosis inducer β-elemene significantly potentiated the efficacy of erlotinib (a first-generation EGFR inhibitor) in EGFR-mutant NSCLC [24]. Consistent with this, our previous study demonstrated that ferroptosis induction represents an effective therapeutic strategy in EGFR 19del NSCLC cells [15]. Recently, osimertinib-tolerant cells were found to exhibit elevated levels of lipid reactive oxygen species and ferrous iron, rendering them more vulnerable to the ferroptosis inducer RSL3 [25]. Together, these findings suggest that triggering ferroptosis may be a viable approach to reverse osimertinib resistance. In this study, we found that HBT significantly induced ferroptosis in osimertinib resistant NSCLC cells, leading to tumor growth repression. HBT also exhibits greater antiproliferative activity against PC9‑OR cells at multiple concentrations (2.5, 5, 15, 30 μM) and shows relatively weaker effects on other NSCLC or normal cells (NCI‑H1299, PC9, RAW 246.7, HMEC‑1). Moreover, HBT treatment leading to PC9-OR cells ferroptosis, as evidenced by lipid peroxidation accumulation, iron overload, mitochondrial morphology and down regulation of GPX4, SLC7A11 and NRF2. These data suggest that HBT may preferentially inhibit resistant cells rather than exerting broad cytotoxicity. Whether this effect specifically reverses acquired osimertinib resistance or simply exploits a ferroptosis vulnerability present in both resistant and parental cells remain to be determined. Future studies using isogenic pairs with or without the resistance mutation will be needed to fully address this question.

Benzothiazoles and their derivatives have garnered increasing interest due to their photoprotective, antioxidant, and antiproliferative properties. As its antioxidant and cytotoxic effect, benzothiazoles and its derivates showed perfect anti-tumor effect in many cancers, such as breast cancer, lung cancer and colorectal cancer [26, 27]. Benzothiazole-based derivatives are known to act as inhibitors targeting multiple key oncogenic players such as EGFR, VEGFR, PI3K, and topoisomerases. Several derivatives, including MKT‑077 and phortress, have advanced to clinical trials to further evaluate their anti-tumor potentia [28–30]. In our recent study, we identified HBT, a benzothiazole derivative isolated from the endophytic fungus *Aspergillus* sp. 1022LEF, which exhibited strong activity against *S. frugiperda* and has been considered a candidate for development as a biopesticide [14]. However, whether HBT can overcome chemotherapy resistance remains unclear. In this study, we report for the first time that HBT effectively reversed osimertinib resistance both in vitro and in vivo, independent of EGFR signaling. Importantly, HBT administration did not significantly affect mouse body weight, major organ morphology, or hepatic and hematopoietic functions. To our knowledge, this is the first report describing the ability of a benzothiazole derivative to overcome chemotherapy resistance. Mechanistic investigations revealed that HBT binds to SCD1 and promotes its degradation, leading to lipid peroxidation and ferroptosis. In summary, HBT represents a promising candidate for treating osimertinib-resistant patients with high SCD1 expression. Our findings also unveil a novel anti-tumor mechanism underlying benzothiazole derivatives, highlighting their potential therapeutic relevance in overcoming drug resistance.

SCD1 is an enzyme that regulate the rate-limiting of monounsaturated fatty acid (MUFA) synthesis. SCD1 is a critical regulator of ferroptosis that can protects cells from ferroptosis by limiting lipid peroxidation [31]. SCD1 is frequently overexpressed in NSCLC and has been implicated in chemotherapy resistance. For instance, SCD1 inhibition overcomes cisplatin resistance in cancer stem cells [32], sensitizes lung cancer cells to ferroptosis inducers such as erastin and RSL3[33], and enhances antitumor T cell responses in NSCLC models [34]. Notably, elevated SCD1 expression has been observed in gefitinib-resistant cells, suggesting its potential role in EGFR tyrosine kinase inhibitor resistance [35]. In this study, we identified HBT as a novel SCD1 inhibitor that can reverse osimertinib resistance by inducing ferroptosis. We showed that HBT‑mediated ferroptosis in PC9‑OR (osimertinib‑resistant) cells is partly dependent on SCD1, suggesting that additional targets may also contribute to its effects. Mechanistically, HBT‑mediated SCD1 suppression likely sensitizes cells to ferroptosis and overcomes osimertinib resistance. These findings collectively underscore SCD1 as a promising therapeutic target for overcoming osimertinib resistance.

In our study, we showed that SCD1 knockdown alone fails to trigger ferroptosis whereas HBT treatment does, likely reflects a threshold effect. In our system, siRNA-mediated knockdown achieved only partial SCD1 reduction, leaving sufficient enzymatic activity to prevent lipid peroxidation. In contrast, pharmacological inhibition by HBT achieved more profound suppression. This interpretation is supported by studies showing that complete SCD1 knockout or strong inhibition effectively induces ferroptosis, while partial knockdown has weaker effects [36, 37]. All these results suggest that SCD1 inhibition is a potential therapeutic strategy for overcoming osimertinib resistance. Furthermore, given that SCD1 has been reported to confer ferroptosis resistance [31, 38], we speculate that HBT treatment may attenuate ferroptosis resistance. And we will further validate it in the future study.

In summary, we discovered HBT, a newly benzothiazoles derivates, as a promising candidate agent to overcome osimertinib resistance. We also revealed novel anti-tumor mechanism of Benzothiazoles-based derivates. Our study suggests that SCD1 is a potential therapeutic strategy for overcoming osimertinib resistance.

## Supplementary Information

Supplementary material 1.

## Data Availability

Data will be made available on reasonable request.
