# Supplementary material for: A fungal-derived benzothiazole derivative overcomes osimertinib resistance in NSCLC by targeting SCD1
Source: Nat Prod Bioprospect. 2026 Sep 11;16(1):86. doi: 10.1007/s13659-026-00645-9 (PMC13569813; doi:10.1007/s13659-026-00645-9)
Supplement: Supplementary file 1 [file 13659_2026_645_MOESM1_ESM.docx]

**Supporting information for**

**A fungal-derived benzothiazole derivative overcomes osimertinib resistance in NSCLC by targeting SCD1**

Boyin Li^1,†^, Yuanyu Ke^2,†^, Jinhua Zhu^3,†^, Weisong Zhang^1^, Mingjin Yang^5^, Zhan Li^1^, Mu Chen^1^, Zhiling Liu^1^, Jieyao Liu^1^, Qiudi Deng^3,*^, Peng Zhang^4,*^, Xueping Lei^1,*^

^1^Guangzhou Municipal and Guangdong Provincial Key Laboratory of Molecular Target & Clinical Pharmacology, the NMPA and State Key Laboratory of Respiratory Disease, College of Pharmacy & The Fifth Affiliated Hospital, Guangzhou Medical University, Guangzhou 511436, PR China

^2^The Second School of Clinical Medicine, Guangzhou Medical University, Guangzhou 510260, PR China

^3^GMU-GIBH Joint School of Life Sciences, The Guangdong-Hong Kong-Macao Joint Laboratory for Cell Fate Regulation and Diseases, Guangzhou Medical University, Guangzhou 511436, PR China

^4^Tobacco Research Institute of Chinese Academy of Agricultural Sciences, Qingdao 266101, PR China

^5^Guangzhou Institute of Industrial Intelligence, Guangzhou 511400, PR China

^*^Corresponding authors.

Email address: dengqiudi@gzhmu.edu.cn (Q.Deng), zhangpeng@caas.cn (P.Zhang), xuepinglei@gzhmu.edu.cn (X.Lei)

^†^These authors contribute equally to the work.

**Supporting Fig.1 The IC50 fold change of osimertinib on PC9-OR, HCC827-OR and NCI-H1975-OR cells.**

**Table S1 Clinical characteristics of 16 NSCLC patients**

| **Number** | **Gender** | **Age** | **Survial (months)** | **Pathological characteristics** | **stage** | **TNM** |
| --- | --- | --- | --- | --- | --- | --- |
| L-0001 | Female | 57 | 125 | LUAD | T1b | IA2 |
| L-0002 | Man | 40 | 78 | LUSC | T1c | IV A |
| L-0003 | Female | 57 | 90 | LUAD | T3 | III A |
| L-0004 | Female | 60 | 38 | LUAD | T1c | II B |
| L-0005 | Female | 71 | 21 | LUAD | T1b | IIIA |
| L-0006 | Female | 54 | 121 | LUAD | T2a | IB |
| L-0007 | Man | 59 | 119 | LUSC | T1b | IA2 |
| L-0008 | Man | 59 | 97 | LUSC | T1b | IA2 |
| L-0009 | Female | 60 | 73 | LUAD | T1a | IA1 |
| L-0010 | Female | 60 | 113 | LUAD | T1a | IA1 |
| L-0011 | Man | 67 | 89 | LUAD | T1c | IV A |
| L-0012 | Man | 59 | 23 | LUSC | T1c | IV A |
| L-0013 | Man | 51 | 32 | LUAD | T2b | II A |
| L-0014 | Female | 60 | 114 | LUAD | T1a | IA1 |
| L-0015 | Female | 57 | 78 | LUSC | T2a | IV A |
| L-0016 | Female | 73 | 14 | LUAD | T2a | IV B |
